# Blocking of ERK1 and ERK2 sensitizes human mesothelioma cells to doxorubicin

**DOI:** 10.1186/1476-4598-9-314

**Published:** 2010-12-15

**Authors:** Arti Shukla, Jedd M Hillegass, Maximilian B MacPherson, Stacie L Beuschel, Pamela M Vacek, Harvey I Pass, Michele Carbone, Joseph R Testa, Brooke T Mossman

**Affiliations:** 1 Department of Pathology, University of Vermont College of Medicine, 89 Beaumont Avenue, Burlington, VT 05405, USA; 2 Department of Medical Biostatistics, University of Vermont College of Medicine, 105 Carrigan Avenue, Burlington, VT 05405, USA; 3 Department of Cardiothoracic Surgery, NYU School of Medicine, 530 First Avenue, 9V, New York, NY 10016, USA; 4 Cancer Research Center of Hawaii, University of Hawaii, 1236 Lauhala Street, 510, Honolulu, HI 96813, USA; 5 Cancer Biology Program, Fox Chase Cancer Center, 333 Cottman Avenue, Philadelphia, PA, 19111, USA

## Abstract

**Background:**

Malignant mesotheliomas (MM) have a poor prognosis, largely because of their chemoresistance to anti-cancer drugs such as doxorubicin (Dox). Here we show using human MM lines that Dox activates extracellular signal-regulated kinases (ERK1 and 2), causally linked to increased expression of ABC transporter genes, decreased accumulation of Dox, and enhanced MM growth. Using the MEK1/2 inhibitor, U0126 and stably transfected shERK1 and shERK2 MM cell lines, we show that inhibition of both ERK1 and 2 sensitizes MM cells to Dox.

**Results:**

U0126 significantly modulated endogenous expression of several important drug resistance (*BCL2, ABCB1, ABCC3*), prosurvival (*BCL2*), DNA repair (*BRCA1, BRCA2*), hormone receptor (*AR, ESR2, PPARγ*) and drug metabolism (*CYP3A4*) genes newly identified in MM cells. In comparison to shControl lines, MM cell lines stably transfected with shERK1 or shERK2 exhibited significant increases in intracellular accumulation of Dox and decreases in cell viability. Affymetrix microarray analysis on stable shERK1 and shERK2 MM lines showed more than 2-fold inhibition (p ≤ 0.05) of expression of ATP binding cassette genes (*ABCG1, ABCA5, ABCA2, MDR/TAP, ABCA1, ABCA8, ABCC2*) in comparison to shControl lines. Moreover, injection of human MM lines into SCID mice showed that stable shERK1 or shERK2 lines had significantly slower tumor growth rates in comparison to shControl lines after Dox treatment.

**Conclusions:**

These studies suggest that blocking ERK1 and 2, which play critical roles in multi-drug resistance and survival, may be beneficial in combination with chemotherapeutic drugs in the treatment of MMs and other tumors.

## Background

Malignant mesotheliomas (MMs), aggressive tumors characterized by marked local invasiveness, are poorly responsive to current therapeutic approaches. Clinical outcomes for MM are poor, resulting in average patient survival times of 7 to 12 months from initial diagnosis. We hypothesized that chemotherapeutic agents used in the treatment of MM activate survival pathways governing drug resistance [1]. For example, abnormal activation of the Raf/MEK/extracellular signal-regulated (ERK) pathway occurs in many human cancers, including MM [2], due to mutations in upstream membrane receptors, Ras and B-Raf, as well as mutations in genes regulating Raf activity that reportedly induces chemoresistance to doxorubicin (Dox) and paclitaxel in breast cancer cells [3]. Moreover, a phase II study in patients with MM shows activation of both ERK and PI3K/AKT pathways that are attributed to their resistance to erlotinib [4].

ERK activation has been identified as a potential survival pathway in several tumor types [5], and recent studies show that ERKs may also be activated in response to chemotherapeutic drugs [6-8] or mTOR inhibitors [9]. We focused here on whether ERK1 and 2 played critical roles in drug resistance and survival of MM, a generally incurable cancer exhibiting marked chemoresistance. To understand the mechanisms involved, we studied gene expression linked to drug resistance and metabolism, including ATP binding cassette (ABC transporters) genes. This large superfamily of membrane proteins is comprised of 48 members that are divided into 7 different families based on sequence similarities [10].

We selected doxorubicin (Dox) (Adriamycin) for our studies as this drug has been widely used as the most successful drug of choice to treat MMs in single agent studies [11,12] and is used currently in treatment of MMs [13,14]. The goal of this study was to understand how Dox-induced resistance develops, and whether it can be overcome by combination therapy. In the present study we demonstrated that Dox treatment causes activation of survival signals (ERK1/2) in MM cells. Combined treatment with a MEK1/2 inhibitor (U0126) plus Dox increased MM cell death over levels observed with Dox alone. Furthermore, using human MM lines expressing shERK constructs, we show that both ERK1 and ERK2 contribute to Dox resistance in human MMs *in vitro *and *in vivo*. Microarray and qRT-PCR analyses of these cell lines revealed that ERK1 or 2 inhibition was linked to decreases in mRNA levels of ATP binding cassette (ABC) genes. Most importantly, we demonstrate that human shERK1 and shERK2 stable MM lines (in comparison to shControl lines) have a slower growth rate after treatment with Dox in a SCID mouse xenograft model. These data suggest that combined treatment using an ERK1/2 inhibitor or RNA interference approach with Dox (or other chemotherapeutic drug) may be more beneficial than single agent therapy in treatment of MMs.

## Methods

### Cell culture

None of the human malignant mesothelioma (MM) lines described in this manuscript are commercially available. However, they have been characterized previously by cell size, doubling time, immunohistochemical analyses, electron microscopy, and chromosomal karyotyping as reported (note that the names of these lines have changed since originally reported)[15]. A sarcomatoid (MO) and epithelioid (ME-26) human pleural MM cell line were obtained from Drs. Luciano Mutti (Maugeri Foundation, Pavia, Italy) and Maurizio Bocchetta (Loyola University, Mayfield, IL), respectively. The HMESO MM line (epithelioid) was originally characterized by Reale et al [16]. PPMMill, a sarcomatoid human MM cell line, was obtained from Dr. Harvey Pass (NYU School of Medicine, New York, NY). Human mesothelial LP9/TERT-1 (LP9) cells, an hTERT-immortalized cell line phenotypically and functionally resembling normal human mesothelial cells [17], were obtained from Dr. James Rheinwald (Brigham and Women's Hospital, Harvard University, Boston, MA). Prior to initiating the studies described here, all isolates were confirmed as MM cells by immunohistochemistry using an antibody to calretinin and verified for lack of mycoplasma contamination using a polymerase chain reaction. Additionally, Hmeso tumor xenografts grown in SCID mice were resected and evaluated immunohistochemically by Dr. Michele Carbone and shown to be cytokeratin positive, indicating that they are mesothelial origin. Subsequent karyotype analysis of the Hmeso line by Dr. Joseph Testa demonstrated that the cells were human and possessed several deletions common in mesothelioma lines. These data support what was originally reported for this MM line [16]. All cells were maintained in 50:50 DMEM/F12 medium containing 10% FBS and supplemented with penicillin (50 units/ml), streptomycin (100 μg/ml), hydrocortisone (100 μg/ml), insulin (2.5 μg/ml), transferrin (2.5 μg/ml), and selenium (2.5 μg/ml), incubated at 37°C in 5% CO_2 _and grown to approximately 80-90% confluency [18]. The synthetic MEK1/2 inhibitor, U0126, and its inactive analog, U0124, were obtained from Calbiochem (La Jolla, CA) and added to cells at 20 μM in medium containing ≤0.2% DMSO [18]. Control cultures received medium without compounds but with vehicle (≤0.2% DMSO) alone and were treated identically. Doxorubicin (Dox) was obtained from Sigma (St Louis, MO).

### Viability determination by cell counting

Viability of cells after Dox treatment was studied by plating cells at 1X10^5 ^per well in a 12 well plate. At confluence, cells were maintained in low serum containing medium (0.5% FBS) for 24 h before treating them with different concentrations of Dox (0-100 μM) for 24 h. Cells were trypsinized and counted using a hemocytometer.

### MTS assay

Human MM cells (transfected or untransfected) were treated with different concentrations of Dox with and without U0126 or U0124 for 24 h, and cell viability was measured in cells using the colorimetric MTS Assay, CellTiter 96 Aqueous One Solution Cell Proliferation Assay (Promega) as per the manufacturer's recommendations. Absorbance was read at 490 nm on a spectrophotometer indicating MTS bioreduction to a colored formazan product by viable cells.

### Western blot analysis

To verify activation of ERK1/2 in MM cells after Dox exposure with and without U0126 or U0124, Western blots were performed as described previously [18] using antibodies specific to pERK1/2 (rabbit polyclonal anti-pERK1/2, 1:500, Cell Signaling Technology, Danvers, MA), total ERK1/2 (rabbit polyclonal anti-ERK1/2, 1:1000, Cell Signaling Technology, Danvers, MA), and total β-Actin 1:2000 (Abcam, Cambridge, MA). Western blots were quantitated by the Quantity One program [18] and normalized to total ERK1/2 levels. Western blotting was also performed to validate the selective inhibition of ERK1 or 2 in sh MM lines.

### Preparation of RNA and PCR array analyses

LP9 and MM cells were grown to confluence and treated with U0126 (20 μM for 24 h). RNA was prepared and purified using a Qiagen RNeasy plus kit (Valencia, CA). After quality assessment, 1 μg of RNA was employed for cDNA synthesis using the RT^2 ^First Strand Kit (SABiosciences, Frederick, MD). Quantitative Real-Time PCR (qRT-PCR) was performed by the Vermont Cancer Center DNA Analysis Facility using RT^2 ^Real-Time™ SYBR Green PCR Master Mix and Human drug resistance and metabolism template RT^2 ^Profiler™ PCR Arrays (SABiosciences) (7900HT Sequence Detection System, Applied Biosystems). Data were analyzed using an on-line spreadsheet-based data analysis template (SABiosciences). qRT-PCR (TaqMan) was used to validate selected genes using Assay on Demand (AOD) Primers and Probes from Applied Biosystems.

### Creation of shERK1 and shERK2 stable MM lines

HMESO cells were selected for these studies because these cells are well-characterized [16] and form MMs reproducibly after injection into SCID mice. Confluent HMESO cells were transfected with either ERK1 or ERK2, or scrambled control Sure Silencing Plasmids (4 sh sequences for each gene were used) from SA Biosciences (Frederick, MD), using Lipofectamine 2000 (Invitrogen, Carlsbad, CA). After selection for 14 days in G418 (400 μg/ml)-containing medium, clones were screened by qRT-PCR for inhibition of ERK mRNA levels as compared to scrambled control (shControl) transfected clones. Two clones from each shERK1 and shERK2 groups were processed by limited dilution to obtain clones in which individual ERKs were inhibited by more than 70% in comparison to shControl clones. Following this procedure, shERK1 and shERK2 clones exhibiting inhibition of >80% ERK expression were obtained. Similarly, shERK1/2 lines were also created from PPMMill lines to verify observations obtained with HMESO line. The experimentally verified shRNA design algorithm (SABiosciences) assures gene-specificity and efficacy. An advanced specificity search in addition to BLAST built into the algorithm helped to reduce potential off-target effects.

### Flow cytometry

To quantitate Dox fluorescence shControl (shCon), shERK1 and shERK2 HMESO cells were grown to confluence and then treated with Dox (0.5 or 5.0 μM) for 1 h or 5 h. Negative controls had no drug added. Cells were washed 3X with phosphate-buffered saline (PBS), trypsinized, counted, suspended in PBS, and Dox fluorescence was examined by flow cytometry using an LSRII flow cytometer (BD Biosciences, MA). A 695/40-nm-band-pass filter with a 685-nm long pass was used to measure Dox fluorescence.

### Fluorescence microscopy for Dox fluorescence

shControl, shERK1 and shERK2 cells were grown to confluence in 4-chambered CultureSlides (BD Falcon, Bedford, MA) in medium containing 10% FBS. Media was replaced with that containing 0.5% FBS 24 h before treatment. Cells were either untreated or treated with 0.5 or 5 μM Dox for 1 h or 5 h at 37°C. Slides with attached cells were then washed in PBS and fixed in 100% methanol for 20 min at -20°C. Slides were washed in PBS and water, allowed to dry, and coverslipped with Aqua Poly/Mount (Polysciences Inc., Warrington, PA). Slides were then stored at 4°C until fluorescent images were acquired using an Olympus BX50 Light Microscope with attached mercury epi-fluorescence illumination.

### Affymetrix gene profiling

Microarrays were performed on MM cell samples from 3 independent experiments as described previously [19]. Each of the samples was analyzed on a separate array, i.e., N = 3 arrays per MM line (3 independent biological replicates). A Human U133A 2.0 array (Affymetrix, Santa Clara, CA) was scanned twice (Hewlett-Packard GeneArray Scanner), the images overlaid, and the average intensities of each probe cell compiled. Microarray data were analyzed using GeneSifter software (VizX Labs, Seattle, WA). This program used a "t test" for pairwise comparison and a Benjamini-Hochberg test for false discovery rate (FDR 5%) to adjust for multiple comparisons. A 2-fold cut-off limit was used to assess statistical significance.

### Quantitative real time PCR (qRT-PCR)

To validate microarray profiles and PCR Array profiles of genes, qRT-PCR (TaqMan) was performed as described previously [19]. Triplicate assays were performed with RNA samples isolated from at least 3 independent experiments. Fold changes in gene expression were calculated using the delta-delta Ct method using hypoxanthine phosphoribosyl transferase (*HPRT1*) as the normalization control. The Assay on Demand primers and probes used were purchased from Applied Biosystems (Foster City, CA).

### Assessment of Dox effects on human tumors appearing after injection of stably transfected shERK1, shERK2 and shControl MM lines in a mouse xenograft model

HMESO cells stably transfected with either shERK1, shERK2 or shControl were injected into 4 subcutaneous sites (5 × 10^6 ^cells per injection site) on the dorsa of 6 wk old Fox Chase Severe Combined Immunodeficient (SCID) mice (Jackson Laboratories, Bar Harbor, ME). All mice (N = 6/group) were weighed weekly and examined every other day for morbidity and tumor growth (measured using a digital caliper). Immediately after tumor appearance (1 wk post cell injection) each group was divided in two subgroups, each containing 3 mice. Three mice in each group were given Dox (6 μg/50 μl saline/tumor site) 3X weekly. The remaining 3 mice from each group received saline (50 μl/tumor site) 3X weekly. The Dox dose and frequency were previously determined to cause no toxicity to mice. After 6 wk of treatment, all mice were weighed and euthanized by intraperitoneal (ip) injection of sodium pentobarbital, necropsied to determine possible gross metastases, and major organs removed and stored in 4% paraformaldehyde before processing for histopathology. Tumors were characterized using previously described histochemical criteria [20] and karyotyped to prove that they were human in origin. Tumor volumes were calculated using formula (π × long axis × short axis × short axis)/6. All experiments using mice were approved by the Institutional Animal Care and Use Committee at the University of Vermont College of Medicine.

### Statistical analyses

In all *in vitro *assays, at least 3 independent samples were examined at each time point per group in duplicate or triplicate experiments. Data were evaluated by ANOVA using the Student Neuman-Keul's procedure for adjustment of multiple pairwise comparisons between treatment groups or using the non-parametric Kruskal-Wallis and Mann-Whitney tests. Differences with p values ≤0.05 were considered statistically significant. The difference in tumor growth rates between different groups in *in vivo *studies was assessed using a hierarchical regression model to take into account the correlation between repeated measurements on the same tumor and multiple tumors in the same animal. In this analysis, the regression coefficient describing tumor growth is modeled as a function of treatment group as well as random variation due to differences between animals and tumors on the same animal.

## Results

### Human MM lines show ERK1 and ERK2 activation in response to low (< LD_50_) concentrations of Dox

**Figure 1:**
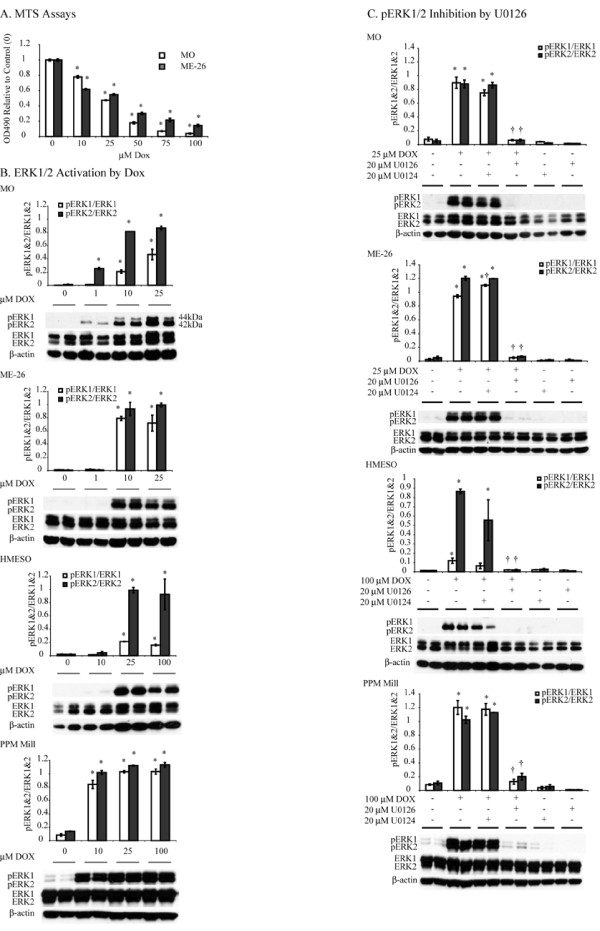
**Human MM lines show ERK1/2 activation after exposures to Doxorubicin (Dox)**. A. Two different MM lines (MO and ME-26) were treated with Dox (0-100 μM) for 24 h and cell viability was determined by the MTS assay as described in the 'Materials and Methods'. *p ≤ 0.05 as compared to control (0), N = 3-6 per group. B. Western blot analysis was performed using specific antibodies for pERK1/2 and total ERK1/2 as described in the 'Materials and Methods'. Both ERK1 and ERK2 were activated (see pERK1 and pERK2) in response to Dox; however, total ERK1 and 2 remained unchanged. *p ≤ 0.05 as compared to respective controls. N = 2 per group. HMESO MM line showed very little ERK1 and pERK1 as compared to other cell lines. C. Dox-activated ERK1/2 in different MM lines is attenuated by the MEK1/2 inhibitor U0126 (20 μM, for 1 h), whereas its inactive analog U0124 is ineffective. *p ≤ 0.05 as compared to respective untreated (0) controls. †p ≤ 0.05 as compared to Dox treated groups. N = 2 per group.

Four MM lines (MO, ME-26, HMESO, PPMMill) were treated with various concentrations (0-100 μM) of Dox for 24 h to determine LD_50 _concentrations. As shown in Figure 1A, a Dox concentration of 25 μM was the approximate LD_50 _concentration for MO and ME-26 lines whereas HMESO and PPMMill lines showed LD_50 _concentrations of approximately 100 μM or greater, respectively (preliminary data not shown). After treatment with various concentrations of Dox, cell lysates were assessed for active (phosphorylated) and total ERK1/2 levels by Western blot analysis. The MO line showed a dose related increase in phosphorylation of both ERK1 and ERK2 that was significant (p ≤ 0.05) starting at the lowest concentrations (1 μM) of Dox used. ME-26 and HMESO lines also showed significant Dox-induced activation of ERK1 and 2 starting at 10 and 25 μM, respectively (Figure 1B), whereas PPMMill cells showed comparable activation of ERK1 and 2 at 10-100 μM Dox. Pre-treatment of human MM cells with the MEK1/2 inhibitor U0126 (20 μM for 1 h) resulted in attenuation of Dox-induced ERK1/2 activation in all MM lines, whereas the inactive analog, U0124 (20 μM for 1 h), had no significant effects on Dox-induced ERK phosphorylation (Figure 1C).

### Dox-induced ERK1/2 activation promotes survival of human MM cells

**Figure 2:**
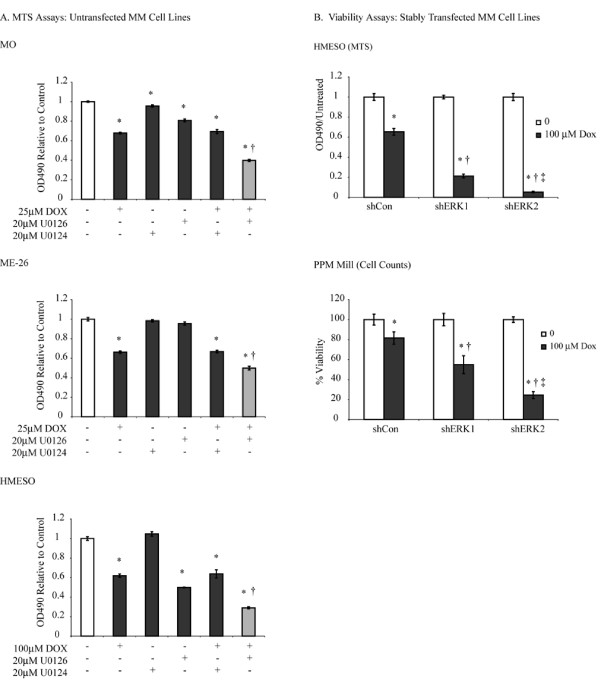
**Dox-induced ERK1/2 activation promotes survival of human MM cells**. A. Three MM lines (MO, ME-26 and HMESO) were pretreated with U0126 or U0124 (20 μM, 1 h) before treating them with an LD_50 _dose of Dox for 24 h. Cell viability was assessed using the MTS assay as described in 'Materials and Methods'. A combined treatment of U0126 and Dox caused significantly increased cell death as compared to either treatment alone (grey bars). *p ≤ 0.05 as compared to untreated control. †p ≤ 0.05 as compared to Dox alone group. N = 3-6 per group. B. HMESO or PPMMill cells were stably transfected with either shERK1 or shERK2 as described in the 'Materials and Methods'. These stable MM lines were treated with Dox (100 μM) for 24 h and cell viability was assessed using the MTS assay or cell counting. Inhibition of ERK1 or ERK2 significantly enhanced Dox-induced cell killing. *p ≤ 0.05 as compared to respective untreated control. †p < 0.05 as compared to Dox treated shControl (shCon). ‡p ≤ 0.05 as compared to shERK1 Dox group. N = 6 per group.

To assess the role of Dox-activated ERK1/2 in cell survival, we pretreated human MM cells (MO, ME-26 and HMESO) with the MEK1/2 inhibitor (U0126, 20 μM) for 1 h before treating for 24 h with Dox at 25 (MO, ME-26) or 100 μM (HMESO), the approximate LD_50 _concentration for each cell type. The MTS assay then was performed to determine cell viability. The higher concentrations of Dox (equal to LD_50 _for various MM cell types) were used for viability assays as lower concentrations of Dox, (1, 5, 25 μM) had no effect on cell viability either alone or in combination with U0126 (data not shown). As shown in Figure 2A (grey bars), treatment with U0126 and Dox resulted in significantly more cell killing in all 3 MM lines evaluated as compared to Dox or U0126 alone. In HMESO and MO cells, U0126 alone also had a significant effect on reducing cell viability, suggesting the possible role of endogenous ERK1/2 activation in cell survival. The inactive analog, U0124 (20 μM for 1 h), had no toxic effects or modulation of Dox-induced cell killing in any MM line, confirming the specific effects of the U0126, MEK1/2 inhibitor.

### Human MM lines have high endogenous expression of many prosurvival and drug resistance related genes which are regulated by ERK1/2

A PCR Array using a 'human cancer drug resistance and metabolism' template on 2 human MM lines (MO, ME-26), compared to the nonmalignant LP9/TERT-1 human mesothelial cell line, showed that both MM lines had significantly (p ≤ 0.05) greater endogenous levels of many prosurvival and drug resistance genes (Table 1 and Additional File 1, Table S1). Of the 10 most highly expressed genes for each line listed in Table 1, mRNA expression of 6 genes (*BCL2, cFOS, MET, BRCA1, ESR2 *and *BRCA2*) was common to both cell lines, whereas 6 genes were differentially expressed. mRNA levels of 2 common genes (*BCL2 *and *cFOS*) highly expressed in each MM line were also validated by qRT-PCR (*p ≤ 0.05 as compared to LP9/TERT-1 cells). In addition to the genes listed in Table 1, many other genes were up-or down-regulated significantly in both cell types and are listed separately in Additional Table 1. Exposure of both MM cell lines to the MEK1/2 inhibitor (U0126, 20 μM for 24 h) resulted in significantly altered levels of some of these genes (*BCL2, cFOS, BRCA1, AR, ESR2, CYP3A4, PPARγ, BRCA2, ABCC3*) (Table 1 and Additional Table 1), suggesting a role of ERK1 or 2 in their regulation.

**Table 1:** PCR Array analysis† showing top 10 endogenously upregulated (p ≤ 0.05) genes in human MM cell lines (MO, ME-26) compared to untransformed LP9/TERT1 mesothelial cells

Gene name (symbol)	Function	Fold Increase (MO)		Fold Increase (ME-26)	
		**-UO126**	**+UO126 20 μM**	**-UO126**	**+UO126 20 μM**

B-cell CLL/Lymphoma (BCL2)*	Drug Resistance	213.65	127.7^§^	28.92	14.57^§^
					
V-fos FBJ murine osteosarcoma viral oncogene homolog (FOS)*	Transcription Factor	26.52	2.28^§^	33.85	20.59^§^
					
Met protooncogene (MET)	Growth Factor Receptor	25.45	28.09	27.60	25.42
					
ATP-binding cassette (MDR/TAP) (ABCB1)	Drug Resistance	13.6	4.66		
					
Estrogen receptor 1 (ESR1)	Hormone Receptors	11.03	10.90		
					
Breast cancer 1, early onset (BRCA1)	DNA Repair	6.66	1.42^‡,§^	6.68	2.88^§^
					
Androgen receptor (AR)	Hromone Receptors	6.35	10.25^§^		
					
Estrogen receptor 2 (ESR2)	Hormone Receptor	4.19	10.48^§^	4.53	6.15
					
Breast cancer 2, early onset (BRCA2)	DNA Repair	3.98	2.21	5.18	2.64^§^
					
Cyclin-dependent kinase inhibitor 2D (CDKN2D)	Cell Cycle	3.68^‡^	4.88		
					
Cytochrome P450 (CYP3A4)	Drug Metabolism			77.12	25.06^§^
					
Peroxisome proliferative activated receptor, gamma (PPARγ)	Hormone Receptor			9.72	4.55^§^
					
Tumor protein 53 (TP53)	Drug Resistance			4.35	4.52
					
ATP-binding cassette (ABCC3)	Drug Resistance			4.56	2.26^§^
					

*Validation by qRT-PCR					

Gene name (symbol)	Function	Validation (MO)		Validation (ME-26)	

		-UO126	+UO126	-UO126 20 μM	+UO126 20 μM

B-cell CLL/Lymphoma (BCL2)	Drug Resistance	550	476	123	31
					
V-fos FBJ murine osteosacroma viral oncogene homolog (FOS)	Transcription Factor	16	2.14	23.88	8.9

^†^SABiosciences: Human drug resistance and metabolism template

^‡^Not significantly different from LP9

^§^Significantly different from -UO126 group of the respective cell line

### Inhibition of either ERK1 or ERK2 sensitizes MM cells to Dox

As the small molecule inhibitor, U0126, abrogated both ERK1 and ERK2 activation, we created stably inhibited (short hairpin, sh) ERK1 and ERK2 HMESO and PPMMill lines to determine if ERKs had similar or unique roles in Dox chemoresistance. The human HMESO and PPMMill MM lines were selected for this purpose as these lines were most insensitive (resistant) to Dox. A significant inhibition (≥70%) of ERK1 or ERK2 in respective lines was obtained as confirmed by Western blotting. In initial *in vitro *experiments, stable shERK1, shERK2 or shControl (shCon) MM lines were treated with Dox (100 μM, ~LD_50 _dose) for 24 h, and cell viability was assessed by the MTS assay (HMESO) or by cell counting (PPMMill). As shown in Figure 2B, shERK1 and shERK2 cell lines showed significantly attenuated cell viability after Dox treatment as compared to shControl lines (Figure 2B, *p ≤ 0.05 as compared to respective untreated control; †p ≤ 0.05 as compared to Dox treated shControl). Although significantly increased Dox-induced cell killing was observed after inhibition of either ERK1 or ERK2, the shERK2 cell lines showed significantly (‡p ≤ 0.05) greater cell killing as compared to the shERK1 lines from both MMs (Figure 2B). The shCon line, as discussed in the 'Material and Method' section, contains a vector with a scrambled sequence, which does not inhibit any gene. shCon cells are expected to behave like untransfected cells as they do in our experiments (compare HMESO in Figure 2A and 2B).

### Inhibition of ERK1 or ERK2 results in greater accumulation of Dox in MM cells

**Figure 3:**
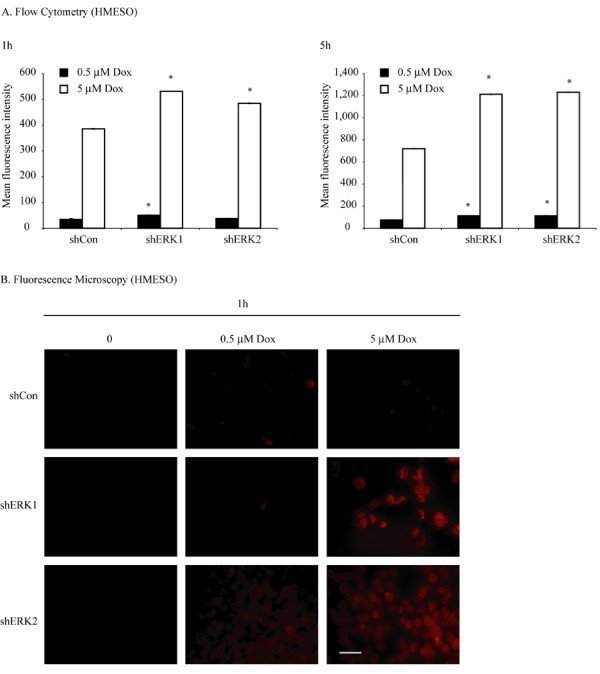
**Inhibition of ERK1 or ERK2 results in more accumulation of Dox in HMESO cells**. A. HMESO cells stably transfected with either shERK1 or shERK2 and shControl cells were treated with 0.5 or 5 μM of Dox for 1 or 5 h, and intracellular accumulation of Dox was measured by flow cytometry as described in 'Materials and Methods'. shERK1 or shERK2 HMESO lines show enhanced accumulation of Dox as compared to shControl lines. *p ≤ 0.05 as compared to respective shControl (shCon). N = 2 per group. B. Fluorescence microscopic representation of dose-related Dox accumulation (orange) in stably transfected HMESO cells after 1 h of treatment. All images are at 400X magnification (Bar = 50 μm).

To show that inhibition of ERK1 or ERK2 increases Dox-induced toxicity by causing greater intracellular accumulation of Dox, we performed flow cytometry experiments on stably transfected HMESO lines treated with Dox (0.5 or 5.0 μM for 1 h or 5 h). Figure 3A shows that MM cell lines stably transfected with either shERK1 or shERK2 exhibited significant dose- and time-related increases in accumulation of intracellular Dox as compared to shControl cells treated with Dox at both time points (*p ≤ 0.05 as compared to respective shControl group). Dox at the low concentration (0.5 μM for1h) was retained marginally but significantly in the ERK1-inhibited (shERK1) HMESO line, whereas high Dox (5 μM for1 and 5 h) was retained by both ERK1 and ERK2-inhibited HMESO (sh) lines as compared to the shCon line treated with Dox. Data in Figure 3A correlate well with findings shown in Figure 2B, where Dox at the high concentration (100 μM for 24 h) shows reduced viability in the shERK2 group. Although Dox retention in both shERK1 and shERK2 groups was similar, the increased toxicity of Dox in the shERK2 group (Figure 2B, HMESO) could be attributed to additional factors. Figure 3B confirms the patterns of Dox accumulation by fluorescence microscopy in MM cells. Note the lack of Dox in the 0 (untreated groups) and the dose-related increases in intracellular fluorescence present in the shERK1 and shERK2 cells.

### Effect of ERK1 or ERK2 inhibition on ATP binding cassette genes (ABC transporters) in MM cells

Based on data above and in Table 1, we next hypothesized that ERKs modulated endogenous expression of ABC cassette genes that function to pump Dox and other chemotherapeutic drugs out of tumor cells, resulting in their decreased drug sensitivity. To address this hypothesis, we performed microarray analysis on shERK1, shERK2 and shControl HMESO cells (no exposure to Dox). Table 2 provides a list of 7 ABC genes (many involved in Dox transport) that had decreased mRNA levels in shERK1 and shERK2 cell lines. Validation of several changes in gene expression was performed using qRT-PCR (*Table 2, p ≤ 0.05 as compared to shControl). We also examined endogenous levels of ABC transporter genes in HMESO MM cells as compared to nontransformed human mesothelial cells LP9/TERT-1 (p ≤ 0.05) (Table 3). These results showed that HMESOs showed striking decreases (> 100 fold) in mRNA levels of *ABCG2 *and *ABCA1 *as well as significant decreases in *ABCA8, ABCC3, ABCB1, ABCG1 *and *ABCC4 *expression, whereas other genes (*MDR/TAP*, *ABCA2*, *ABCC5 *and *ABCA7*) were upregulated.

**Table 2:** Microarray analysis showing endogenous expression of ATP-binding cassette genes in shERK1 and shERK2 cells compared to shControl, stable HMESO cells (p ≤ 0.05)

Gene name(symbol)	Fold Change shERK1 vs. shCon	Fold Change shERK2 vs. shCon
ATP-binding cassette, subfamily G (WHITE), member 1 (ABCG1)	-4.89	
		
ATP-binding cassette, subfamily A (ABC1), member 5 (ABCA5)*	-2.77	
		
ATP-binding cassette, subfamily A (ABC1), member 2 (ABCA2)*^†^	-2.13	
		
Transporter 2, ATP-binding cassette, subfamily B (MDR/TAP)†	-2.09	
		
ATP-binding cassette, subfamily A (ABC), member 1 (ABCA1)	-2.06	
		
ATP-binding cassette, subfamily A (ABC1), member 8 (ABCA8)*		-2.38
		
ATP-binding cassette, subfamily C (CFTR/MRP), member 2 (ABCC2)*^†^		-2.19

*Validation by qRT-PCR		

Gene name(symbol)	Fold Change shERK1 vs. shCon	Fold Change shERK2 vs. shCon

ABCA2	-2.88	
ABCA5	-3.6	
ABCA8		-3.4
ABCC2		-3.0

† Indicates Doxorubicin is a substrate as reported in the literature.

**Table 3:** Microarray analysis showing endogenous levels of ATP-binding cassette genes in HMESO MM cells as compared to normal mesothelial cells (LP9/TERT-1) (p ≤ 0.05)

Gene symbol	Fold Change
ABCG2	-125
ABCA1	-102
ABCA8	-16
ABCC3	-8
ABCB1	-3.8
ABCG1	-2.2
ABCC4	-2.1
Transporter 2, ABCB (MDR/TAP)	3.4
ABCA2	2.7
ABCC5	2.5
ABCA7	2.5

### Tumors developing from shERK1 and shERK2 MM lines in a mouse xenograft model show decreased tumor growth rate after treatment with Dox

**Figure 4:**
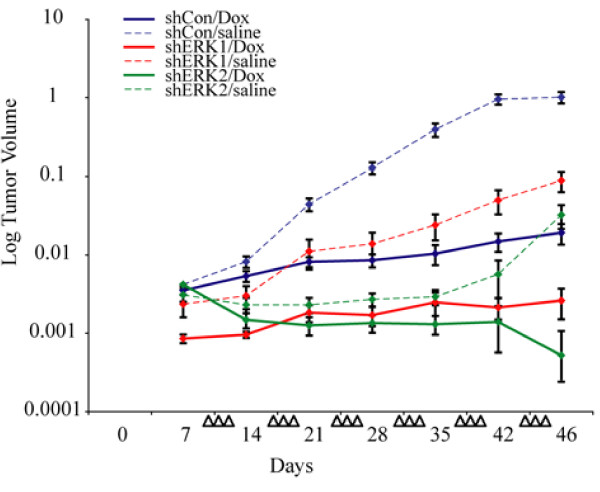
**ERK inhibited tumors in a mouse xenograft model show enhanced sensitivity to Dox**. HMESO cells (5 × 10^6^) stably transfected with shERK1, skERK2, or shControl (shCon) constructs were injected subcutaneously at 4 dorsal sites on SCID mice. Eight days after cell injections, localized Dox or saline injections (6 μg/50 μl/tumor, 3 times a wk for 6 wks) were started (shown by arrow heads). At autopsy, tumors were harvested and volumes were calculated as described in 'Materials and Methods'. N = 3 mice/group, or 12 tumors/group. Statistical analysis (see Materials and Methods) performed showed that Dox-treated mice in the shERK1 or shERK2 groups had significantly slower tumor growth than the Dox-treated mice in the shControl group. All conclusions were derived by statistical analysis (described in the Method section) performed on different groups to compare alteration in tumor growth rate and not tumor volume.

To verify the functional effects of ERK inhibition and Dox treatment on tumor cell killing, we injected stable shERK1, shERK2 or shControl HMESO MM cells subcutaneously into SCID mice, and treated various groups with Dox or saline at the tumor site as soon as tumors appeared (1 wk post cell inoculation, 3X weekly) for a 6 wk period. As shown in Figure 4, Dox significantly reduced the rate of tumor growth in all three animal groups compared to saline treatment, with the largest reduction occurring in the shControl group. In addition, Dox-treated animals in the shERK1 or shERK2 groups had significantly slower tumor growth than the Dox-treated animals in the shControl group. The differences between the shControl Dox-treated and shERK1 Dox-treated tumor growth rates occurred prior to 21 days post MM cell injection. All conclusions were derived by statistical analysis (described in the Method section) performed on different groups to compare alterations in tumor growth rate and not tumor volume.

## Discussion

Treatment of various MM lines with doses of Dox much lower than LD_50 _concentrations resulted in phosphorylation of ERK1 and 2, the most abundant ERKs in mammalian cells. In addition to Dox, many other anti-cancer drugs such as paclitaxel and cisplatin induce activation of ERKs in different tumor types [21-23]. However, taxol inhibits ERK activation in different cell types depending upon experimental conditions [23]. In our study, Dox-induced ERK1/2 activation protected MM cells from Dox-induced cell death, as shown when MM lines were pretreated with the MEK1/2 inhibitor, U0126, prior to Dox exposure (Figure 2). In support of our findings, it has been reported that, in most cases, ERK activation protects cells from drug-induced cell death [23,24], while in some tumor cells, ERK activation contributes to cell death [21,22]. These different effects may be explained by differences in subcellular distribution of specific ERKs, the longevity of ERK signaling, or phosphorylation of different substrates which may dictate death or survival [25].

We studied 4 different MM lines for Dox responses after ERK1/2 manipulation either with an inhibitor (U0126) or by shRNA approaches. With the use of the ERK1/2 inhibitor (inhibits both ERK1 and 2), HMESO cells were the best responders (most susceptible) as compared to MO and ME-26 (both lines showed the same susceptibility) (Figure 2A). A shRNA approach to inhibit either ERK1 or ERK2 was studied in 2 MM lines (HMESO and PPMMill). Of the two lines studied by this approach, HMESO again showed more sensitivity to Dox-induced killing after ERK1 or ERK2 inhibition as compared to PPMMill (Figure 2B). In addition, in both cell lines, ERK2 inhibition was more effective than ERK1 inhibition in Dox-induced cell killing (Figure 2B).

Although regulation of apoptotic pathways has been implicated in resistance of many cancers to chemotherapy, we show that human MM lines endogenously overexpress many prosurvival genes (*BCL2, cFOS, MET*, etc.) in comparison to nontransformed mesothelial cells. The increased levels of these commonly upregulated genes, as reported by our lab and others [18,26-29] may in part be responsible for drug resistance in MM cell lines. For example, *BCL2 *and *BCL-xL *antisense treatment facilitates apoptosis in mesothelioma cells, suggesting *BCL2/BCL-xL *bispecific antisense treatment in combination with cisplatin or gecitabine may result in a more effective therapy of MM [30]. Consistent with our findings, ERK1/2 activation has been linked to expression and activation of *BCL2 *in various systems [3,31] resulting in an anti-apoptotic or survival outcome. *cFOS*, a protooncogene and component of activator protein-1 (AP-1), is upregulated by crocidolite asbestos in rat pleural mesothelial cells [32], and endogenously upregulated in human mesothelioma cell lines and tumors [18,28]. We show for the first time that *BRCA1 *and *BRCA2 *are endogenously overexpressed in MM cells, and are pursuing their mutation and functional status in various MMs. ERK1/2 has been linked to feedback regulation of the tumor suppressor/DNA repair gene *BRCA1 *in irradiation induced DNA damage checkpoint activation [33,34]. *BRCA2 *was also endogenously upregulated in MM cells and ERK1/2 inhibition decreased expression of this gene (Table 1), consistent with already published work that ERK1/2 activation inhibits replication of prostate cells via upregulation of *BRCA2 *[35]. Another gene, PPARγ, which was upregulated only in ME-26 and was significantly inhibited by the U0126 MEK1/2 inhibitor is activated via an ERK1/2 dependent COX-2 pathway in macrophages [36]. Inflammatory pathways involving PPARγ or COX-2 are promising therapeutic targets in a number of cancers [37]. We also report for the first time the upregulation of a cytochrome P450 enzyme gene, CYP3A4, related to drug metabolism in the ME-26 epithelioid cell line that was decreased 3-fold after addition of U0126. The presence of the androgen receptor and its endogenous expression in sarcomatoid MM cells is also a novel finding, and both AR and ESR2 have been linked to the ERK pathway [38-40] as shown in Table 1 in MO cells. A recent study suggests that ER-β affects the prognosis of MM by acting as a tumor suppressor [41].

ATP-binding cassette (ABC) transporters transport various molecules, including chemotherapeutic drugs, across extra- and intracellular membranes. Increased expression of one or more of these proteins is seen in almost all resistant cancers and is considered responsible fully or in part for the observed drug resistance in most cancer cell lines. In a previous study using MM cell lines, coordinated overexpression of the multi drug resistance pump (MRP) and gamma-glutamylcysteine synthetase genes, but not MDR1, correlated with Dox resistance [42]. In the 3 MM lines we studied by PCR array or microarray analysis, different types of ABC transporter genes were endogenously overexpressed as compared to untransformed LP9/TERT-1 mesothelial cells (Table 1, Table 3). The overexpression of different types of ABC genes in different MM cells further confirms the highly heterogenic nature of MM tumors that vary widely in their prognosis and response to therapy. Inhibition of ABC genes by ERK1 or 2 inhibition may be responsible for the increased accumulation of Dox observed in shERK1 and shERK2 MM cells (Figure 3). Among ABC genes inhibited by shERK2 in HMESO cells, *ABCA8 *is a relatively uncharacterized new transporter [43] whereas Dox is a known substrate for *ABCC2*, *ABCA2 *and *MDR/TAP *[44-46]. Our data suggest that different ERKs regulate distinct ABC genes, and a detailed study is needed to understand the roles of different ERKs, including ERK5 that has been linked to chemoresistance in breast cancers [47], in ABC gene regulation. Consistent with our studies, ERK1 and 2 are linked to regulation of many ABC genes, including *ABCG1*, *ABCA1*, *MDR1*, and *MRP1 *in various cancer and non-cancer cells [6,48-50].

## Conclusions

Our *in vitro *and *in vivo *studies here indicate that both ERK1 and ERK2 play significant roles in imparting Dox resistance to MM cells by modulating genes related to drug resistance and survival previously unidentified in MM cells. Most importantly, we demonstrate that gene expression of distinct ABC transporters is modulated by blocking ERK1 or ERK2, and show the relationship of these phenomena to Dox accumulation in human MM cells. Further, we demonstrate that blocking ERK1 and ERK2 enhances the chemotherapeutic potential of Dox in a murine xenograft model. The mechanisms of ERK1/2 action appear to involve both upregulation of prosurvival/antiapoptotic genes as well as ABC transporter genes. Based on our observations, ERK1/2 inhibitors in combination with chemotherapeutic drugs might be a better option to treat patients with MM than drugs alone.

## Competing interests

The authors declare that they have no competing interests.

## Authors' contributions

AS and BTM designed research; AS, JMH, MBM and SLB performed research; HIP contributed new reagents and tools; AS, BTM and PMV analyzed data; and AS and BTM wrote the paper. AS, JMH, MBM, SLB, BTM, PMV, MC, HIP and JRT critically reviewed the manuscript and given final approval of the version to be published.

## Supplementary Material

Additional file 1 **Supplemantal Table 1**. PCR array analysis showing significantly (p ≤ 0.05) up or down regulated^† ^genes (≥2-fold) in human MM cell lines (MO. ME-26) with and without the U0126 MEK1/2 inhibitor (U0126, 20 μM) compared to untransformed LP9/TERT1 mesothelial cells. Click here for file
