# Supplementary material for: Blocking of ERK1 and ERK2 sensitizes human mesothelioma cells to doxorubicin
Source: Mol Cancer. 2010 Dec 15;9:314. doi: 10.1186/1476-4598-9-314 (PMC3016286; doi:10.1186/1476-4598-9-314)
Supplement: Additional file 1 — Supplemantal Table 1. PCR array analysis showing significantly (p ≤ 0.05) up or down regulated† genes (≥2-fold) in human MM cell lines (MO. ME-26) with and without the U0126 MEK1/2 inhibitor (U0126, 20 μM) compared to untransformed LP9/TERT1 mesothelial cells. [file 1476-4598-9-314-S1.PDF]

Supplemental Table 1. PCR Array analysis* showing significantly ($p \leq 0.05$) up or down regulated† genes (≥ 2-fold) in human MM cell lines (MO, ME-26) with and without the U0126 MEK1/2 inhibitor (U0126, 20μM) compared to untransformed LP9/TERT1 mesothelial cells

	MO		ME-26	
Gene Symbol	- U0126	+ U0126	- U0126	+ U0126
ABCB1	13.6	4.66		
ABCC1	-2.32		2.14	3.6
ABCC2	-4.14	-2.3	-2.89	-2.04
ABCC3	-3.62	-3.33	4.56	2.26
ABCC6	-6.69	-6.86	-2.81	
ABCG2	-9.29	-19.05	3.2	5.26
AHR				-2.34
AP1S1	2.23	2.38		
AR	6.35	10.25	-3.76	
ARNT				2.19
ATM	-3.2	-2.7		
BAX		2.75		
BCL2	213.65	127.71	28.92	14.57
BCL2L1	2.87	4.14		2.33
BLMH		2.26		2.27
BRCA1	6.66		6.68	2.88
BRCA2	3.98	2.21	5.18	2.64
CCND1				-2.72
CCNE1	2.92	3.75		2.63
CDK2	3.06		2.64	2.83
CDK4				2.78
CDKN1A		3.69	-11.89	-4.08
CDKN1B				5.47
CDKN2A	-49.84	-44.39	-81.39	-84.97
CDKN2D		4.88		5.22
CLPTM1L			3.05	3.71
CYP1A1		3.19	10.86	8685.44
CYP2D6			2.63	2.11
CYP2E1	3.31	5.77	3.93	3.78
CYP384		2.73	77.12	25.06
CYP3A5		-2.19		
DHFR		-4.46	4.03	2.4
EGFR		2.64	2.5	2.3
ELK1		2.04		3.85
EPHX1	-4.4		2.55	3.52
ERBB2				
ERBB3	-7.46	-5.26	-2.15	
ERBB4				4.68
ESR1	11.03	10.9	3.13	2.08
ESR2	4.19	10.48	4.53	6.15
FGF2				-4.35
FOS	26.52	2.28	33.85	20.59
GSTP1				2.11
IGF1R				-2.64

IGF2R			2.04	2.21
MET	25.45	28.09	27.6	25.42
MSH2			2.51	2.73
MYC				2.5
NAT2	-34.55	-47.41	-117.95	-93.71
NFKB1			2.23	
NFKB2		4.35	2.26	3.63
NFKBIE	2.18	3.62	3.9	11.77
PPARD				
RARA				
RARB	-46.34	-109.57	-83.67	-195.86
RARG	2.63	3.34		2.35
RB1			2.55	2.22
RELB	2.47	9.36	2.78	5.88
RXRA				3.12
SOD1		-2.03		
SULT1E1	-178.84	-945.64	-14.13	-170.06
TNFRSF11A			2.33	
TOP1			4.33	5.4
TOP2A			2.37	2.62
TOP2B			3.29	2.84
TP53			4.35	4.52
XPA			2.02	2.21
XPC	-3.68	-2.27		

* Human drug resistance and metabolism template was used
† Negative values represent downregulated genes
